# The reliability of plantar pressure assessment during barefoot level walking in children aged 7-11 years

**DOI:** 10.1186/1757-1146-5-8

**Published:** 2012-03-20

**Authors:** Stephen D Cousins, Stewart C Morrison, Wendy I Drechsler

**Affiliations:** 1 School of Environmental and Life Sciences, Faculty of Engineering, Health, Science and the Environment, Charles Darwin University, Darwin 0909, Australia; 2 School of Health, Sport and Bioscience, University of East London, England, Stratford E15 4LZ, UK

**Keywords:** Plantar pressures, Gait, Paediatrics, Reliability

## Abstract

**Background:**

Plantar pressure assessment can provide information pertaining to the dynamic loading of the foot, as well as information specific to each region in contact with the ground. There have been few studies which have considered the reliability of plantar pressure data and therefore the purpose of this study was to investigate the reliability of assessing plantar pressure variables in a group of typically developing children, during barefoot level walking.

**Methods:**

Forty-five participants, aged 7 to 11 years, were recruited from local primary and secondary schools in East London. Data from three walking trials were collected at both an initial and re-test session, taken one week apart, to determine both the within- and between-session reliability of selected plantar pressure variables. The variables of peak pressure, peak force, pressure-time and force-time integrals were extracted for analysis in the following seven regions of the foot; lateral heel, medial heel, midfoot, 1st metatarsophalangeal joint, 2nd-5th metatarsophalangeal joint, hallux and the lesser toes. Reliability of the data were explored using Intra Class Correlation Coefficients (ICC 3,1 and 3,2) and variability with Coefficients of Variation (CoV's).

**Results:**

The measurements demonstrated moderate to good levels of within-session reliability across all segments of the foot (0.69-0.93), except the lesser toes, which demonstrated poor reliability (0.17-0.50). CoV's across the three repeated trials ranged from 10.12-19.84% for each of the measured variables across all regions of the foot, except the lesser toes which demonstrated the greatest variability within trials (27.15-56.08%). The between-session results demonstrated good levels of reliability across all foot segments (0.79-0.99) except the lesser toes; with moderate levels of reliability reported at this region of the foot (0.58-0.68). The CoV's between-sessions demonstrated that the midfoot (16.41-36.23%) and lesser toe region (29.64-56.61) demonstrated the greatest levels of variability across all the measured variables.

**Conclusions:**

These findings indicate that using the reported protocols, reliable plantar pressure data can be collected in children, aged 7 to 11 years in all regions of the foot except the lesser toes which consistently reported poor-to-moderate levels of reliability and increased variability.

## Background

Plantar pressure assessment is commonly used in the clinical evaluation of the foot and provides insight into the plantar loading characteristics during functional activities such as walking and running [1]. This data can be incorporated into the assessment and evaluation of foot and lower limb function and to enhance management planning and treatment objectives [2]. Normative plantar pressure data has been reported in the assessment of the typically developing child [3] and has advanced our understanding on the loading of the foot during developmental stages [4,5] and to evaluate paediatric foot deformity in disease specific populations [6].

The use of pressure assessment in practice is beneficial, however, gait in young children is idiosyncratic and collection of reliable data is challenging [7]. Commonly reported variables among the literature include the peak measures of pressure and force during the assessment of the loading characteristics of the feet in children [8-10]. However, it has been recommended that these measures alone do not give sufficient information with regards to the overall loading characteristics of the feet and should be quoted alongside temporal parameters such as pressure-time and force-time integrals, which will give an indication as to the effects of the peak loading values on the soft-tissue and joint structures of the feet [11].

Reliable pressure and gait data for children can be affected by a number of developmental variables, such as foot structure and gait maturation. It has been acknowledged in the literature that by the ages of 6-7 years the major structural changes have been completed in the child's foot, giving it a similar appearance to that of an adult's foot [12,13]. It has also been acknowledged that children exhibit characteristics of gait maturation from 3 years of age as evidenced by the presence of a reciprocal arm swing, heel strike and toe-off, increased walking velocity, step length and single support coupled with a reduction in cadence [14,15]; with maturation complete by the ages of 6-8 years [16]. However, recent studies have implied that gait maturation may continue beyond the age of 8 years and may not be complete until 13 years of age [17,18]. This research indicates that it is prudent to consider specific age ranges when testing children rather than a wide range across developmental levels.

There are numerous commercially available systems currently employed by clinicians and researchers alike to assess plantar loading [19]. The reliability of equipment commonly used for methods of plantar pressure assessment has been established in a normal population of adults [20-23], however subsequent work using a paediatric population is lacking. Early work by Hughes et al. [21], reported a good level of reliability was achieved in ten adults, for force and pressure variables, across twelve regions of the foot, with the reliability of all measurements increasing with the number of trials analysed. Hughes et al. [21] also reported measurements related to time were more variable than the peak measures of force and pressure. Recently, Gurney et al. [23] conducted a study looking at the reliability of plantar pressure measurement in an adult population. Nine adults were recruited into this study and it was concluded that areas of the foot where high loads were experienced resulted in greater reliability (ICCs > 0.9) when compared to areas with lesser loading (ICCs < 0.8). This work is in agreement with that of Zammit et al. [20] who also reported moderate-to-good reliability (ICC's, 0.51-0.95), in thirty healthy adults, for peak force and pressure through seven regions of the foot, during barefoot level walking.

Whilst the work of the previously mentioned authors is of interest, it is important to acknowledge that the direct extrapolation of this work to the paediatric population may be invalid. It is commonly recognised in clinical practice that children's gait is associated with increased variability and therefore it is necessary to establish the feasibility of repeatable plantar pressure measurement in this population as the value of this clinical assessment is as yet undetermined.

At present, to the authors' knowledge, no study has sought to investigate the reliability of protocols to assess plantar pressure measurements in a paediatric population. Lack of this analytical approach, whilst attempting to assess the measures, will lead to doubts regarding the usefulness of such data for clinical and research purposes. Therefore, the aim of this study was to recruit children aged 7 to 11 years to determine the reliability in assessing plantar pressure measurements, using the variables of Peak Pressure, Peak Force, Pressure-time Integral and Force-time Integral across two test sessions.

## Methods

### Participants

Prior to the recruitment of participants the Ethics Committee at the University of East London, London, England provided ethical approval for the study (**ETH/08/94/0)**. Following this, information sheets and consent forms were distributed amongst local schools.

The sampling pool for this study consisted of one primary and two secondary schools based in East London, where the ages of the pupils ranged from 4 to 16 years. Parental consent and child assent was obtained prior to data collection and forty-five (n = 45) typically developing children, aged 7 to 11 years were recruited. The 7 to 11 year age banding was considered appropriate for this study, in order to negate the influence of developmental factors on gait and foot loading characteristics. The forty-five children recruited into this study represents nine participants within each of the age groups; with previous studies using similar numbers when investigating the reliability of repeated plantar pressure measurements in a healthy population of adults [21,23].

Children were excluded from participation if they disclosed a history of orthopaedic, neurological and/or musculoskeletal problems likely to affect their gait.

### Measurement apparatus

The MatScan^® ^3150 pressure distribution platform (TekScan, USA) was used for the collection of all dynamic trials. This system consists of a 5 mm floor mat composed of 2,288 resistive sensors, with a resolution of 1.4sensors/cm^2^, a sensor matrix measuring 439.5 mm by 369.9 mm and a sampling frequency of 40 Hz (Hz).

### Procedure

To capture dynamic plantar pressures the midgait protocol was used, this method involves striking the platform with at least the fourth step to ensure a constant velocity has been reached prior to contact with the platform. This protocol allows a more accurate reflection of a subjects' gait in comparison to abbreviated gait protocols, such as the first-step and two-step methods. These protocols having been shown to significantly reduce peak pressures and forces beneath the feet during walking and subsequently, the data obtained during these trials may not be generalisable to normal walking conditions [24,25]. Also, given the suggestion that steady state gait is not achieved until the end of the second or third step [26-28], gait protocols should ideally involve a minimum preamble of at least three steps if representative gait patterns are to be obtained.

All subjects were given time to familiarise themselves with the process of walking over the platform to ensure they were comfortable with the experimental procedure. During data collection the subjects were encouraged to adopt a natural gait pattern and to walk at a self-selected speed. No attempt was made to control the speed of the subjects as previous work with children demonstrated that a less than natural gait was observed when precise gait instructions were given [29]. Trials were excluded and repeated if a participant targeted the platform, altered their gait pattern to ensure full contact with the mat, if the participant paused on the mat whilst walking, or if the participant did not continue to walk past the mat with at least five steps. Three complete trials of the right foot were recorded for each participant, this number of trials has previously been found to be sufficient in ensuring adequate reliability of force and pressure data [21,30]. To satisfy assumptions of data independence [31], data from the dominant foot was collected and this was the right foot for all participants [31]. Plantar pressure measurements were recorded at baseline and repeated one week later.

**Figure 1:**
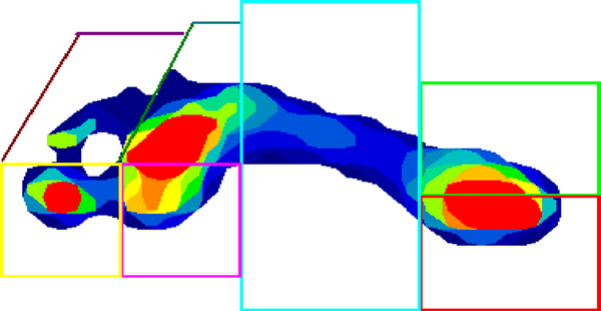
**An example of a typical walking trial produced by the TekScan MatScan^® ^system, displaying the seven masked regions used during analysis**.

The peak measures of pressure (kPa) and force (N) alongside the temporal measures of pressure-time integrals (kPa.s/cm^2^) and force-time integrals (N.s/cm^2^) were selected for this research study. These variables are commonly used during the assessment of foot loading in children [8-10]. Following data collection, MatScan^® ^Research Software version 6.4 was used to construct individual foot masks to determine the plantar loading characteristics under seven discrete regions of the foot: lateral heel, medial heel, midfoot, 1st metatarsophalangeal joint (1MPJ), 2nd-5th metatarsophalangeal joint (2-5MPJ), hallux and the lesser toes (Figure 1) to provide detailed information regarding the plantar loading of different segments of the foot. There is no current consensus in this literature regarding the definition of foot segments, but for the purpose of this study a similar template was used to previous studies investigating the reliability of plantar forces and pressures during barefoot level walking in healthy adults [20] and to examine age-related changes in foot function [32]. Once this template had been performed on an individual footprint it was then saved and used repeatedly for analysis on further trials (for each participant). All footprints were masked by the lead author (SDC) who applied the same foot mask across all trials for each participant. A single rater was chosen to perform all the manual masking so as to reduce the potential for introducing variability into the data, with previous research showing good reliability of a manual mask application being possible when performed by a single rater [20,33].

### Statistical analysis

Statistical analysis was conducted using SPSS version 15.0 for Windows (SPSS Inc, Chicago, USA). Prior to inferential analysis all data was tested for normality using the Kolmogoroff-Smirnoff one-sample test. In all cases the variables were normally distributed and reliability was evaluated using the mean of three trials. Calculation of the mean occurred by summating the values, then dividing by the number of trials [34].

Within-session reliability was determined from the original testing session for the 45 subjects using repeated measure ANOVA's to calculate Intraclass Correlation Coefficients (ICC, model 3,1). For all the measured variables across all regions of the foot ICC model 3,1 was selected for analysis. Variability in the data were assessed via the calculation of Coefficients of Variation (CoVs); this analysis of absolute reliability provides information regarding within-trial variability expressed as a percentage.

To assess systematic differences between sessions, paired t-tests were used to compare the mean values of all the measured variables across each discrete region of the right foot, with statistical significance being defined as p < 0.05. Between-session reliability was evaluated using both relative reliability statistics (ICC model 3,2) derived from repeated measure ANOVA's and absolute reliability statistics (mean difference and CoVs) using the mean of three trials derived from each testing session. Interpretation of the within- and between-session ICCs (3,1 and 3,2) was conducted in accordance with Portney and Watkins [34] whereby values of > 0.75 indicate good reliability, values ranging from 0.5 to 0.75 imply moderate reliability and values < 0.5 suggest poor reliability. CoVs provide information regarding between-trial variability expressed as a percentage thus enabling direct comparisons between variables measured in different units. The mean difference was also calculated to provide an indication of the overall change in the scores that occurred between each testing session.

## Results

### Participant characteristics

The mean ± SD (range) for the age and height of the participants (n = 45) was 9.00 ± 1.43 (7-11) years and 1.37 ± 0.09 (1.19-1.71) metres respectively. The weight and BMI values reported for the participants were 32.61 ± 9.57 (20.01-100.18) kg and 17.04 ± 3.41 (12.32-34.08) kg/m^2^. Males comprised 60% (n = 27) of the overall sample.

### Within-session reliability

All within-session measures of reliability are reported in Table 1. The parameters demonstrated moderate-to-good within-session reliability, ranging from 0.69-0.93 for all of the variables across all segments of the foot except the lesser toes, which demonstrated poor reliability (0.17-0.50) for each of the measured variables. CoVs across the three repeated trials ranged from 10.22-27.15% for peak pressure, 10.95-41.67% for peak force, 13.87-48.31% for pressure-time integrals and 13.37-56.08% for force-time integrals in children aged 7 to 11 years. In all cases the lesser toe region demonstrated the greatest variability within trials for all four parameters.

**Table 1:** Within-session reliability (Intraclass correlation coefficients [ICC] and coefficients of variation [CoV]) obtained from three repeated trials

Region	Peak pressure		Peak force		Pressure-time integral		Force-time integral	
	ICC (95% CI)	CoV (%)	ICC (95% CI)	CoV (%)	ICC (95% CI)	CoV (%)	ICC (95% CI)	CoV (%)
Lateral heel	0.90 (0.82-0.96)	10.22	0.88 (0.77-0.95)	13.44	0.91 (0.86-0.97)	14.62	0.81 (0.61-0.86)	17.33
Medial heel	0.83 (0.68-0.90)	12.55	0.90 (0.83-0.94)	12.74	0.88 (0.69-0.92)	13.87	0.88 (0.69-0.93)	17.01
Midfoot	0.85 (0.70-0.92)	15.97	0.71 (0.59-0.82)	17.92	0.83 (0.75-0.86)	19.33	0.76 (0.43-0.80)	18.80
1MPJ	0.73 (0.60-0.89)	12.88	0.82 (0.67-0.92)	19.21	0.85 (0.79-0.88)	15.61	0.81 (0.62-0.86)	15.47
2-5MPJ	0.92 (0.84-0.95)	12.52	0.93 (0.82-0.95)	10.95	0.74 (0.51-0.87)	19.84	0.92 (0.75-0.98)	13.37
Hallux	0.74 (0.56-0.88)	13.85	0.71 (0.50-0.86)	18.66	0.69 (0.45-0.84)	17.11	0.69 (0.43-0.73)	17.96
Lesser toes	0.50 (0.29-0.67)	27.15	0.47 (0.18-0.62)	41.67	0.46 (0.15-0.61)	48.31	0.17 (0.03-0.23)	56.08

### Between-session reliability

The relative and absolute measures to determine the between-session reliability are reported in Tables 2, 3, 4 and 5. The only region of the foot to display a significant mean difference between testing sessions was the lesser toes for the variables of peak pressure (p < 0.05), peak force (p < 0.05) as well as pressure-time (p < 0.05) and force-time integrals (p < 0.05).

**Table 2:** Between-session reliability of peak pressure (kPa)

Mean of three trials					
Region	Session 1 (Mean ± SD)	Session 2 (Mean ± SD)	Mean Difference	ICC (95% CI)	CoV (%)
**Lateral heel**	268.21 ± 54.78	267.68 ± 41.50	0.53	0.96 (0.92-0.98)	6.81
**Medial heel**	228.08 ± 60.65	225.91 ± 58.15	2.17	0.97 (0.93-0.99)	10.68
**Midfoot**	102.75 ± 71.17	99.22 ± 71.35	3.53	0.99 (0.96-0.99)	16.41
**1MPJ**	149.17 ± 43.97	147.52 ± 47.38	1.65	0.97 (0.89-0.99)	15.24
**2-5MPJ**	202.25 ± 66.74	203.37 ± 61.41	-1.12	0.93 (0.79-0.95)	8.50
**Hallux**	194.28 ± 63.38	188.39 ± 57.94	5.89	0.93 (0.83-0.96)	11.26
Lesser toes*	58.32 ± 26.30	88.06 ± 67.83	-29.74	0.67 (0.48-0.87)	29.64

* significant difference (p < 0.05) between session 1 and 2

**Table 3:** Between-session reliability of peak force (N)

Mean of three trials					
Region	Session 1 (Mean ± SD)	Session 2 (Mean ± SD)	Mean Difference	ICC (95% CI)	CoV (%)
**Lateral heel**	754.36 ± 154.25	746.81 ± 133.62	7.55	0.96 (0.92-0.99)	22.83
**Medial heel**	621.98 ± 149.51	624.71 ± 152.33	-2.73	0.98 (0.94-0.99)	24.78
**Midfoot**	305.96 ± 193.37	308.33 ± 226.65	-2.37	0.79 (0.58-0.84)	26.86
**1MPJ**	411.56 ± 108.78	416.07 ± 107.05	-4.51	0.95 (0.91-0.99)	23.45
**2-5MPJ**	602.79 ± 182.58	601.25 ± 170.36	1.54	0.87 (0.67-0.96)	23.38
**Hallux**	549.81 ± 158.01	582.27 ± 142.47	-32.46	0.83 (0.61-0.91)	25.99
Lesser toes*	130.12 ± 69.12	128.44 ± 71.45	1.68	0.62 (0.23-0.73)	51.82

* significant difference (p < 0.05) between session 1 and 2

**Table 4:** Between-session reliability of pressure-time integrals (kPa.s/cm^2^)

Mean of three trials					
Region	Session 1 (Mean ± SD)	Session 2 (Mean ± SD)	Mean Difference	ICC (95% CI)	CoV (%)
**Lateral heel**	28.84 ± 2.94	31.12 ± 3.96	-2.28	0.98 (0.91-0.99)	12.67
**Medial heel**	27.48 ± 3.25	28.33 ± 4.15	-0.85	0.87 (0.77-0.95)	11.41
**Midfoot**	14.60 ± 2.55	16.45 ± 2.28	-1.82	0.99 (0.93-0.99)	18.14
**1MPJ**	24.82 ± 3.35	26.08 ± 2.65	-1.26	0.89 (0.80-0.98)	10.13
**2-5MPJ**	32.67 ± 3.90	31.65 ± 3.41	1.02	0.93 (0.85-0.98)	11.08
**Hallux**	27.25 ± 3.65	28.83 ± 3.18	-1.58	0.96 (0.88-0.98)	11.50
Lesser toes*	9.04 ± 3.26	10.01 ± 3.09	-0.97	0.68 (0.47-0.72)	31.75

* significant difference (p < 0.05) between session 1 and 2

**Table 5:** Between-session reliability of force-time integrals (N.s/cm^2^)

Mean of three trials					
Region	Session 1 (Mean ± SD)	Session 2 (Mean ± SD)	Mean Difference	ICC (95% CI)	CoV (%)
**Lateral heel**	410.72 ± 110.76	434.63 ± 94.63	-23.91	0.93 (0.89-0.96)	25.04
**Medial heel**	247.56 ± 121.41	249.11 ± 104.17	-1.55	0.93 (0.90-0.96)	26.78
**Midfoot**	323.05 ± 164.97	350.56 ± 159.83	-27.51	0.83 (0.63-0.89)	36.23
**1MPJ**	283.09 ± 88.86	289.75 ± 84.02	-6.66	0.95 (0.82-0.98)	29.54
**2-5MPJ**	437.23 ± 151.63	431.07 ± 98.26	6.16	0.82 (0.63-0.86)	23.71
**Hallux**	200.55 ± 44.23	208.33 ± 39.04	-7.78	0.90 (0.85-0.94)	21.85
Lesser toes*	45.23 ± 25.01	58.21 ± 11.13	-12.98	0.58 (0.25-0.69)	56.61

* significant difference (p < 0.05) between session 1 and 2

Good levels of between-session reliability (> 0.79) were reported for all variables across all foot segments except the lesser toes. The between-session results also demonstrated moderate levels of reliability at the lesser toe region of the foot (0.58-0.68). The CoVs between-sessions demonstrated that the lateral heel (6.81-25.04%), medial heel (10.68-26.78%), 1MPJ (10.13-29.54%), 2-5MPJ (8.50-23.71%) and the hallux (11.26-25.99%) reported the least variability between the data sets; whereas the midfoot (16.41-36.23%) and lesser toe region (29.64-56.61%) demonstrated the greatest levels of variability across all the measured variables.

When comparing the mean of three measurements across two testing sessions, mean differences ranged from -29.74-5.89 kPa for peak pressure, -32.64-7.55 N for peak force, -2.28-1.02 kpa.s/cm^2 ^for pressure-time integrals and -27.51-6.16 N.s/cm^2 ^for force-time integrals.

## Discussion

Plantar pressure assessment is commonly used to provide insight into the plantar loading characteristics of the paediatric foot. Due to their common use in both a clinical and research setting, it is necessary to ensure that the protocols for plantar pressure assessment in children can reproduce plantar pressure measures of dynamic foot function on different occasions. To date no study has also considered the reliability of protocols for the assessment of plantar pressure data in typically developing children and therefore, the purpose of this study was to determine the reliability of plantar pressure variables in a group of children aged 7 to 11 years, during barefoot level walking.

The results demonstrated that the collection of reliable plantar pressure variables is possible in children for all foot segments except the lesser toes. The within-session ICCs for the seven analysed regions of the foot ranged from 0.50 to 0.92 for peak pressure, 0.47 to 0.93 for peak force, 0.46 to 0.91 for pressure-time integrals and 0.17 to 0.92 for force-time integrals. All variables, except at the lesser toes recorded consistently moderate-to-good levels of reliability, whereas the lesser toe region reported poor levels of reliability, particularly for the force-time integral (0.17). However, the ICC is a unitless value and does not provide an indication of absolute variability and therefore further analysis using CoVs (%) was conducted. This analysis showed a similar pattern to the ICCs in that the reliable variables demonstrated smaller CoV percentages (10.22 to 19.84%) for all foot segments in comparison to the lesser toes (27.15 to 56.08%). These results indicate that within a single testing session, repeated measurements at the lesser toes are associated with reduced reliability and increased variability during gait, in comparison to the other six regions of the foot.

Assessment of systematic differences between sessions indicated that peak pressure, peak force, pressure-time integral and force-time integral at the lesser toe region exhibited a significant mean difference between sessions (p < 0.05). The remaining six regions, across all four variables, did not display any systematic differences between sessions, captured one week apart. Relative reliability between sessions was consistently good, with all regions of the foot, except the lesser toes reporting ICC values greater than 0.79. The lesser toe region again reported lower reliability (0.58-0.68) across the four variables. The between-session CoVs for the seven analysed regions of the foot ranged from 6.81 to 29.64% for peak pressure, 22.83 to 51.83% for peak force, 10.13% to 31.75% for pressure-time integrals and 21.85 to 56.61% for force-time integrals. Due to the absence of an agreed upon criteria for the assessment of CoV values, it is difficult to comment upon the acceptability of the values derived from this study. However it is important to note that the CoV's reported in this study are typically higher for the four measured variables across the seven regions of the foot, in comparison to those reported in the investigation into the reliability of repeated plantar pressure measurements in adults [20,23] and may indicate an increased variability in the foot loading patterns of children during gait.

The midfoot and lesser toe region displayed the greatest percentage difference for all four variables, highlighting the greater variability within different regions of the foot. The findings at the midfoot and lesser toe regions in this study are consistent with previous reports in adults [20,23] and indicate that these regions of the foot may be subject to inherent variability during gait. The reduced reliability between sessions at the lesser toes reported in this study are also consistent with those reported by Gurney et al. [23]. Findings from the adult study were that reliability was reduced in areas of the foot where loading was less typical, in this instance it was the lateral lesser toes (defined as the 3rd-5th toes). Also of interest, the authors reported reduced reliability at the medial mid-foot (ICC < 0.8) which was not noted in the present study. Although these results are of interest direct comparison between studies is difficult due to differences in the participants, methodologies adopted and in the sensor technology used. The study by Gurney et al. [23] assessed reliability using the Novel EMED^® ^plantar pressure platform, in comparison to the TekScan MatScan^® ^system used in this study. The Novel EMED^® ^platform used by Gurney et al. [23] has a slightly higher spatial resolution of 2 sensors/cm^2 ^in comparison to 1.4 sensors/cm^2 ^of the TekScan MatScan^® ^system; it also has a slightly higher sampling frequency of 50 Hz in comparison to 40 Hz respectively. Consequently, although the collection of reliable plantar pressure data appears possible in children, the lower spatial resolution of the TekScan MatScan^® ^system may limit the validity of this system in accurately being able to isolate small regions of the foot, such as the lesser toes and highlights the importance of the resolution of a system when assessing the plantar pressures in children with small foot sizes.

The work by Gurney et al. [23] highlighted that the definition of foot segments is not uniform or consistent across different studies. Gurney et al. [23] divided the mid-foot into lateral and medial midfoot whereas the present study defined the mid-foot as one segment. The definition of the foot segments between studies limits direct comparison. Furthermore, the methods used for segmental division of the foot vary between systems and can be subject to error resulting from the sensor resolution of the platform and anatomical knowledge of the observer's involved [35]. Recently, Deschamps et al. [33] conducted a study looking at the reliability of manually determined masks across the forefoot in plantar pressure footprints and concluded that that the masking of small segments of the foot be conducted with caution. This is in agreement with the work of Latour et al. [36] and Urry and Wearing [22] whom both commented that the lower reliability at the lesser toes may be due to limitations with sensor technology in isolating this small region of the foot, particularly in young children.

Normative pressure data have been published by Alvarez et al. [3] who identified age-related differences in plantar pressure profiles in a sample of 146 children ranging from 1.6 years-14.9 years. In their study the foot was divided into five segments however the lesser toes were not considered separately, rather as part of the lateral forefoot. They reported that comparable foot pressure profiles could be identified across the age groups which were: (i) children under the age of two years; (ii) children aged two-five years and (iii) children older than five years. This work is interesting because it suggests that whilst there is debate in the literature regarding the development of a mature gait [7], the loading characteristics of children over five years are consistent and can yield reliable data. However, with the limitation of the lesser toes being incorporated as part of the lateral forefoot, further work is required to explore the reliability of this specific region of the foot in children.

Plantar pressure assessment is complex and challenging in young children and it is important to acknowledge that the capture of reliable data is dependent on a number of different factors, such as the participants (age, pathology and developmental status), instrumentation (sensor technology and validity of instrumentation) and the adopted protocols (abbreviated vs. midgait protocols, gait velocity). Factors such as walking speed have been reported to affect pressure variables [37] and where appropriate should be taken into consideration. To the authors' knowledge, there are no studies which have attempted to standardise or control cadence during plantar pressure studies in children and thus would suggest that standardisation may not be appropriate in the child. However, this is an area that warrants further investigation.

There are several limitations to this present study that need to be considered when interpreting the findings. First, only typically developing children were recruited, so the reliability of these measurements cannot be generalised to a clinical population. Confounding variables such as pain or asymmetrical gait commonly reported in symptomatic populations may have a significant impact upon the reproducibility of plantar measurements taken one week apart. Secondly, Zammit et al. [20] has previously discussed limitations with the use of the TekScan MatScan^® ^including factors such as the manual masking procedure for the determination of plantar pressure outputs for each individual as well as the relatively low sampling frequency (40 Hz) and spatial resolution (1.4 sensors/cm^2^). All of which may affect the validity and reliability of repeated measures and in accurately isolating small regions of the foot as seen in children. Finally, the results from this study can only apply to the age group under investigation. Further work is required to investigate the reliability of plantar pressure measurements in a younger group of children, due to the potential influence of the continuing development of their foot structure. Additional research would also be required to confirm these results in an older cohort of children due to any influence postural changes have upon musculoskeletal development during puberty and adolescence.

The present study has demonstrated that reliable plantar pressure data can be collected in children aged 7-11 years, however it must be acknowledged that the results presented can only apply to the sample under investigation and to the instrumentation used in this study. Further work is required to explore the reliability of plantar pressure data collection across children of varying ages and postural development to determine the factors which influence the reliability of plantar pressure data capture. There is also a need to explore the reliability of plantar pressure assessment where deformity is present.

## Conclusion

In this study, we have presented protocols for the capture of plantar pressure variables in a sample of typically developing children. This work demonstrated that the collection of reliable plantar pressure data within a single session and between two sessions is possible in children. The results suggest that most segments of the foot yield reliable data for the four analysed variables of peak pressure, peak force, pressure-time integrals and force-time integrals with the exception of the lesser toes which warrant further investigation.

## Abbreviations

1MPJ: 1st metatarsophalangeal joint; 2-5MPJ: 2nd-5th metatarsophalangeal joint.

## Competing interests

None of the authors have any financial or personal relationships with other people or organisations that could inappropriately influence this work.

## Authors' contributions

SDC, SCM and WID all conceived and designed the study. SDC collected and analysed the data. SDC drafted the manuscript with the assistance of both SCM and WID. All three authors approved the final manuscript.
